# Importance of Genetic–Fitness Correlations for the Conservation of Amphibians

**DOI:** 10.3390/ani13223564

**Published:** 2023-11-18

**Authors:** Heike Pröhl, Ariel Rodríguez

**Affiliations:** Institute of Zoology, University of Veterinary Medicine of Hannover, Bünteweg 17, 30559 Hannover, Germany; ariel.rodriguez@tiho-hannover.de

**Keywords:** genetic diversity, heterozygosity, fitness, infection, amphibian

## Abstract

**Simple Summary:**

Threatened animal species live in small and isolated populations, often with a reduced number of individuals and reduced genetic diversity. In this review, we summarise studies on amphibian species that examined the effect of genetic diversity on the fitness of individuals or populations. We found evidence for the positive association between genetic diversity and different estimates of fitness where low genetic diversity is associated with lower fitness. Published studies are however scarce, concentrated on a handful species and with a high proportion of non-significant results reported. For the maintenance of amphibian and other animal populations, it is important to increase the research effort in this topic and, for imperilled populations, enhance genetic diversity through different types of conservation measures.

**Abstract:**

Endangered animals suffer from isolation of their habitats. Isolation leads to a reduction in population size as well as a decrease in genetic diversity and a concomitant increase in the risk of extinction. Amphibians are the most endangered vertebrate class. Besides habitat loss, fragmentation and isolation, amphibians are threatened by emerging diseases e.g., chytrid fungus or *Ranavirus*. By employing experiments, researchers investigate whether changes in genetic diversity within or among isolated populations affect amphibian fitness. While genetic diversity estimates are based on molecular markers, typically microsatellites, fitness is mostly measured as tadpole performance in rearing experiments often under varying environmental conditions. Tadpole performances (e.g., body mass, growth rate and survival) have been found to be negatively affected by low genetic diversity, as several studies have found a positive association between genetic diversity and these fitness traits. Moreover, infection with pathogens also seems to be more likely in individuals or populations with lower genetic diversity. Overall, these genetic–fitness correlations seem to be more pronounced or detectable in smaller, declining populations but not in larger populations. Genomic studies, which sample a larger fraction of the genome, are still scarce in the conservation genetic literature on amphibians. These are likely to increase in upcoming years and may reveal adaptive variants that protect against dangerous pathogens or environmental changes. Altogether, genetic–fitness correlation studies should be a priority in order to develop effective management plans for the genetic rescue of isolated, imperilled amphibian populations.

## 1. Introduction

Genetic diversity is highly important to maintain healthy animal and plant populations and assure their long-term persistence [1,2]. If populations are isolated and small, several genetic problems might arise. First, genetic drift might lead to a random fixation and loss of alleles, which depletes the genetic variation required for ecological adaptation to changing environments. At the same time, genetic load induces the accumulation of deleterious alleles because selection is less efficient in small populations. Moreover, if the number of potential mates is restricted in a small population the risk of inbreeding—mating among relatives—rises. Inbreeding increases the number of homozygous loci including loci with deleterious, partially recessive mutations. Inbreeding depression is the consequence: it is a serious risk for declining populations because it impairs fitness traits such as birth weight, survival probability, fecundity and resistance to diseases [3,4,5].

The classical study by Reed and Frankham [6] has evidenced that genetic diversity (in the following text abbreviated as ***GD***) in vertebrates, invertebrates and plants correlates significantly with population fitness and decreases when population sizes decline. For example, in deer mice from nine populations of Michigan, parasite load (nematode *Capillaria hepatica*) is a proxy for fitness that negatively correlates with ***GD*** (measured as allozyme heterozygosity) [7]. However, the consequences of decreased ***GD*** for the species and their conservation are not always clear. Populations of some species seem to persist or even grow although their ***GD*** is low (e.g., *Bufo calamita* [8], or *Alces alces* [9]; reviewed in [10]), while others suffer from inbreeding depression due to small population size and genetic load (e.g., [11,12,13]).

Despite some discrepancies in the research results, the role of ***GD*** as well as certain genetic factors is important for the persistence of populations in many taxa [14,15]. The introduction of individuals into threatened populations would enhance ***GD*** and reduce their chance of extinction resulting in a genetic rescue [16,17]. ***GD*** has been traditionally measured with neutral molecular markers such as microsatellites [18]; however, more recently the focus of research has been switched to the genomic level, a much better representation of the total genetic variation. Genomic approaches provide fine-scale genetic data for multiple loci distributed across all parts of the genome or even covering the whole genome [19].

Amphibians are particularly vulnerable to anthropogenic activities that often result in declines in population sizes. Compared to other vertebrates, a higher proportion of amphibian species (amphibians: 41%, birds: 13%) are endangered [20,21]. Amphibian populations suffer from land-use conversion, leading to habitat fragmentation and destruction as well as the contamination and spread of infectious diseases [22,23]. A total of 36 out of the more than 7486 amphibian species are already extinct or possibly extinct (156), and many other species (>3314) are affected by population declines [21]. Habitat fragmentation reduces gene flow among populations and consequently depletes genetic variation. Higher genetic distances (F_ST_ values) indicating population fragmentation have been found for amphibians (F_ST_ = 0.32, N = 33 amphibian species) than for other taxa (e.g., F_ST_ = 0.076 for birds and F_ST_ = 0.26 for reptiles, [2]). For example, studies on the yellow-bellied toads *Bombina variegata* in Germany revealed highly structured populations with pairwise F_ST_ values which were significantly different among most populations and an overall extremely low genetic diversity (as measured by heterozygosity, H_O_, H_E_ and inbreeding F_IS_) in most toad localities [24,25]. Other examples for highly structured amphibian species with low ***GD*** are the Oregon spotted frog *Rana pretiosa* from the Pacific Northwest [26], and the Arouss Al Ayn *Salamandra inframaculata* from northern Israel [27]. 

The reduction in amphibian population sizes interacts with decreases in ***GD***, population bottlenecks and inbreeding. Together these challenges result in fitness reductions, especially under stressful conditions (reviewed in: [28,29]). These facts pose an increasing problem for amphibian conservation. Therefore, this review aims to summarize the published evidence on the genetic–fitness correlations (GFC) in amphibian species. We consider studies that estimated ***GD*** at neutral as well as adaptive loci. Some of these studies also found particular alleles associated with fitness. Our work reveals that GFC indeed exists in many species, but that the proxies used to evaluate fitness as well as the methods to estimate ***GD*** have been diverse. We discuss how to standardize the methods, the need to increase the number of amphibian species assessed and finally the overall importance of GFC for conservation management in highly imperiled species.

## 2. Material and Methods

**Figure 1:**
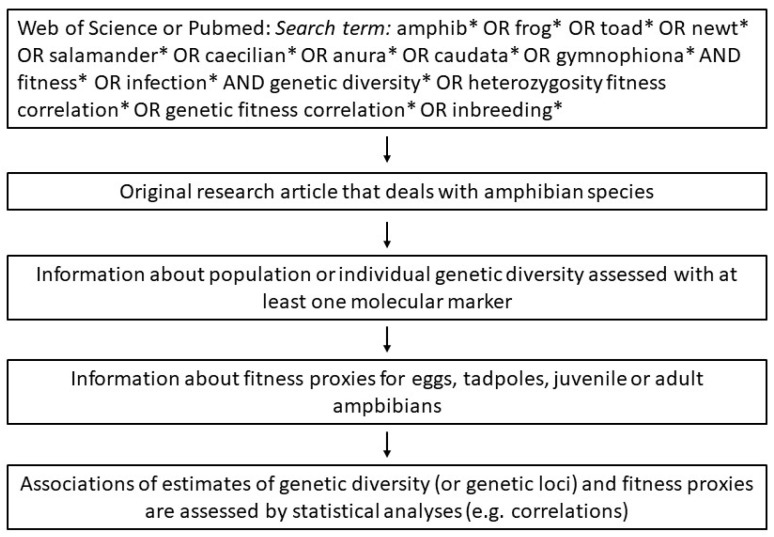
Decision flow for including publications in this review. The * is a Boolean search operator that matches any alphanumeric string.

For this review, we searched for literature on amphibian species that report correlations between genetic variation and fitness (*GFC*) or heterozygosity and fitness (*HFC*). Therefore, we used the global citation databases Web of Science and PubMed in February 2023. We used the following key words for the search: amphib* OR frog* OR toad* OR newt* OR salamander* OR caecilian* OR anura* OR caudata* OR gymnophiona* AND fitness* OR infection* AND genetic diversity* OR heterozygosity fitness correlation* OR genetic fitness correlation* OR inbreeding*. From all articles displayed (Web of Science: N = 379 and PubMed: N = 262), we read the abstract and methods to decide whether the article contained the desired information for the topic. In total, we found 35 articles that met our criteria. The criteria were as follows: estimates of genetic diversity (***GD***) and a statistical test of association with fitness traits of an amphibian species were reported (Figure 1). To summarize information and results from the publications, we elaborated a detailed table with the following content: species, study area (country), number of sample sites investigated, genetic method used to estimate ***GD***, estimators of ***GD*** (e.g., allelic richness), the proxies for fitness (e.g., tadpole survival or infection intensity with the chytrid fungus *Bd*), the applied statistical method, the presence or absence of correlations or associations between ***GD*** or certain genetic elements (e.g., immune genes) with the fitness proxies, and finally a short summary or conclusion from the study. We additionally report—only in cases where this information was available in the publication—the population size of the focal species at the sample site and values (or ranges) of genetic diversity estimates. In cases where the genetic population structure has been evaluated, we report the number of genetic clusters found (K) via Bayesian Assignment algorithms (e.g., STRUCTURE) and F_ST_ values. In experiments or when differences in the environment in the field have been measured, we shortly outline the treatments or ecological factors. Since the studies differed in the genetic markers used and analytical procedures, the results (e.g., heterozygosities) among studies cannot be directly compared. In most studies the ***GD*** measures are reported for populations (Ho or He), in some studies however ***GD*** and fitness were measured on the individual level (e.g., MLH or *Bd* infection intensity). This is then indicated as “indiv.” genetic variation. If available, we transferred values of correlations (r or R2) and probabilities (P) from the publication into the table. However, since the statistical models applied are highly diverse among studies this information could not be standardised. Since the information content in the table is complex we simplified the content by dividing it into three tables. The first table covers the genetic markers used to measure ***GD*** and how different fitness parameters are affected by ***GD*** and certain adaptive loci. The results of the experiments which analysed the effects of ecological conditions on fitness parameters and how these interact with ***GD*** and those that analyse the effect of small population size and/or population isolation on amphibian fitness were also tabulated.

To graphically summarise the compiled data we aggregated the diversity of the phenotypic variables measured in the original articles into eight main fitness trait categories as follows: development (1/tadpole deformities, tadpole development rate and egg hatch success), growth rate (tadpole growth rate and juvenile growth rate), fertility (adult reproductive investment and clutch size), health (Bd tolerance, 1/Bd infection and 1/Bd intensity), home range, size (tadpole mass, tadpole size, adult size and tadpole weight), and survival (tadpole survival, egg survival, clutch survival, juvenile survival and adult survival). We then registered the reported statistical effect of the ***GD*** on these fitness traits (positive, negative or non-significant) and the amphibian species studied in each case. To summarise the phylogenetic diversity of the studied species, we used a comprehensive amphibian phylogeny [30] pruned to only include the studied species using the *phytools* package [31].

## 3. Results

### 3.1. Study Overview

**Figure 2:**
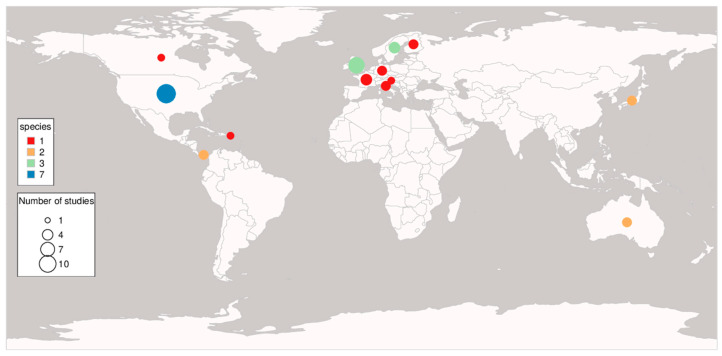
Distribution of studies reporting genetic diversity of populations or individuals and correlations to fitness-related traits in amphibians.

In total we found 35 studies published in a diversity of international journals that contained information about ***GD*** and at least one fitness-related trait for one up to 38 study sites (SS) in different amphibian species (Table S1). Most studies only included one species, two studies included two species [32,33] and one study included six species [34]. The later study, however, tested for the association of GD and fitness traits between, but not within, species and hence is not directly comparable to the rest of studies. One study included two closely related *Atelopus* species, but based on the genetic population structure analysis it was concluded that no clear genetic distinction between the two species was possible and therefore they should be considered one species [35]. In total, 33 studies considered anura, one considered caudata, one considered one anura and one caudata species, but no caecilian species was considered in the publications. Twenty-four studies were realised in the temperate zone, seven in the subtropical zone and four in the tropical zone. The species discussed were eleven temperate species, seven subtropical species and nine tropical species. The mismatch between “studies per climate zone” and “species per climate zone” is due to the fact that several temperate and one subtropical species were included in several publications: for example, the temperate species *B. calamita* and *Rana temporaria* were investigated five times and *Hyla arborea* four times, while none of the tropical species were included in more than one study (Figure 2, Table S1). We excluded the study of six Brazilian [34] species from Figure 1 and Figure 2 as well as Table 1 because information on the genetic diversity and fitness was not available at the species level. However, the study is still included in Table S1 and last table since the relationship between forest fragmentation and *Bd* infection is relevant.

The genetic markers involved were diverse. Genotyping microsatellites (N = 24) were the obvious preferred method in the lab; however, allozymes (N = 2), minisatellites (N = 1), RAPD (N = 1), AFLPs (N = 1), Cytb (N = 2) and unspecified mtDNA (N = 1) also served as measures of neutral genetic variation. Both Cytb studies were performed in combination with microsatellites. Eight studies used an adaptive immunological locus, the MHC IA or II β (exon 1 or 2) loci, four of those also used microsatellites, one combined with mtDNA, and two also used a genomic approach. Furthermore, five studies used genomic approaches: four studies sequenced SNPs (single nucleotide polymorphisms), while one study applied transcriptome sequencing (Table S1). The measures of ***GD*** depended on the molecular marker used: the most important ones for microsatellites are allelic richness (AR), observed H_O_ and expected heterozygosity H_E_ for populations as well as multilocus heterozygosity (MHL) for individuals. Allele frequencies and heterozygosity, gene diversity (π) and Tajimas D (T*D*) were also reported for MHC results, and the three CytB/mtDNA studies reported gene diversity (π) and/or Tajimas D (Table S1). Several studies comprised information about the population structure: global F_ST_ values are given in three studies [36,37,38], while pairwise/mean pairwise F_ST_ values are given for four studies [39,40,41,42]. Interestingly, the non-isolated population in *Rana latastei* from Italy showed much smaller pairwise F_ST_ values than the isolated populations [39]. Accordingly, for *R. temporaria* in Sweden, the global F_ST_ value for a fragmented study area was more than three times higher than the global F_ST_ value for a continuous habitat [37]. In five studies the population structure analyses were also applied using Bayesian cluster analysis with the program STRUCTURE [43] or *NGSadmix* [44]. The number of identified genetic clusters ranged from K = 2 in *Anaxyrus boreas* [45] to K = 10 in *Litoria verreauxii* [46]; thus, all amphibian species analysed showed genetic population structuring. Twenty studies were conducted on egg and/or tadpole stages, while fourteen studies included adult individuals and one study included both juveniles and adults (Table S1).

### 3.2. Genetic Diversity and Fitness

Measurements of ***GD*** have been conducted in all but one study (*H. arborea*; [47]), but the values for ***GD*** have been reported in only 22 studies (Table S1). The study on *H. arborea* without genetic diversity analysis compared the fitness of genetically small and isolated populations with large populations and was therefore included in this review. Fourteen studies found evidence for a positive effect of high ***GD*** on fitness traits or a negative effect of low ***GD*** (e.g., [48]; Table 1). Twelve studies found evidence for a positive effect of ***GD*** on one or several fitness traits but no effect on other fitness traits (e.g., [49]). Six studies found no effect of ***GD*** on fitness traits (e.g., [47]). Only one study found positive and negative effects of ***GD*** on fitness traits: in *R. temporaria,* tadpole survival and developmental rates were positively correlated with ***GD,*** while growth rate was negatively correlated [50]. Two studies found *Bd* infection to be higher in genetically more diverse populations [45,51], while May et al. [52] found the same relationship between neutral genetic diversity and *Bd* infection; however, they found no effect of ***GD*** on tadpole traits and that adaptive MHC diversity was higher in uninfected populations. None of the studies found either no effect or negative effects of ***GD*** on fitness traits. In summary, 26 studies found positive effects of ***GD*** on fitness traits in amphibians, while only four studies found a negative relationship between ***GD*** and fitness proxies, and this involved *Bd* infection in three cases (Table 1). Additionally, eight studies found an association between certain MHC loci or certain candidate genes related to survival and/or *Bd* infection (Table 1).

In terms of the measured fitness traits investigated in all studies, 35 measured fitness traits were positively correlated with ***GD*** or negatively affected by particularly low ***GD***, while 34 fitness traits showed no relationship with ***GD*** and four fitness traits (e.g., lower or no *Bd* infection) were associated with lower ***GD***. Since some studies involved two or more molecular markers, it is possible that fitness traits were correlated with the ***GD*** measured at one marker but no correlation was found for ***GD*** measured with another marker (N = 3, [34,36,53]). For example, in *Bufo bufo* tadpoles, survival and deformity were significantly associated with allozyme diversity but not with minisatellite diversity [36].

**Figure 3:**
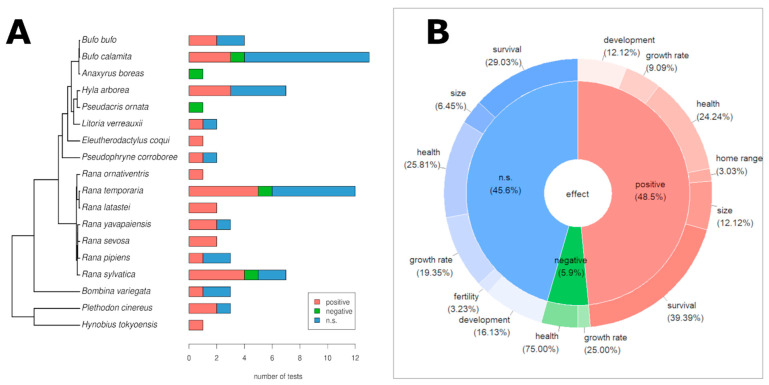
Graphical summary of the association between genetic diversity and fitness traits of amphibians obtained from published studies. (**A**) Stacked bar plots of the number of positive, negative and non-significant associations reported for the species studied. (**B**) Doughnut chart summarizing the proportions by fitness trait categories and statistical association results across all species.

We compiled a total of 69 statistical tests of association between ***GD*** and fitness traits in 18 species. Four species (*B. calamita*, *H. arborea*, *R. temporaria* and *R. sylvatica*) were the most studied and accounted for 57% of all the statistical tests conducted. Overall, the majority of the reported ***GD*** effects on fitness traits were positive (>48% positive vs. 6% negative), although the number of non-significant effects reported was high overall (>45%) (Figure 3).

**Table 1:** Studies examining the association between fitness traits and calculated genetic diversity (***GD***) or a certain genetic locus (below) evaluated with different genetic markers. Note that a fitness trait can be positive, i.e., enhancing fitness (e.g., growth rate, survival) or negative, i.e., decreasing fitness (e.g., the presence of disease or deformity). Cases in which the expectation that higher ***GD*** is positively correlated with “positive” fitness is not met are marked with 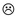. Those studies that contain information about ***GD*** and genetic loci associated with fitness appear twice in the table.

Species	Genetic Marker /Locus	Fitness Trait Correlated with *GD*	Correlation	Fitness trait Negatively Affected When Genetic Diversity Is Low	Fitness Traits Not Correlated with *GD* or Not Affected by Low *GD*	Citation
*Bufo bufo*	allozymes minisats	tadpole survival tadpole deformity	positive negative		tadpole survival tadpole deformity	[36]
*Bufo calamita*	microsats	tadpole growth rate	positive	egg hatch rate	tadpole survival	[49]
*Hyla arborea*	allozymes	--	--	--	egg hatch rate tadpole survival	[54]
*Bufo calamita*	microsats	--	--	--	tadpole survival tadpole growth rate tadpole develop. rate tadpole time to metamorf.	[32]
*Rana temporaria*	microsats	--	--	--	tadpole survival tadpole growth rate tadpole develop. rate tadpole time to metamorf.	[32]
*Bufo calamita*	microsats			tadpole survival tadpole growth rate		[48]
*Rana temporaria*	microsats	tadpole survival tadpole develop. rate tadpole growth rate	positive positive negative 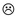			[50]
*Rana latastei*	microsats	tadpole survival	positive			[55]
*Rana sylvatica*	microsats	sibship survival (eggs—tadpole stage)	positive		tadpole weight	[56]
*Rana sylvatica*	RAPD	egg survival tadpole survival tadpole deformity	positive positive negative			[57]
*Rana latastei*	microsats	egg hatch rate	positive			[39]
*Rana temporaria*	microsats	tadpole body size tadpole survival	positive positive			[37]
*Rana temporaria*	microsats	tadpole weight	positive		age at metamorphosis	[58]
*Bufa calamita*	AFLPs	tadpole survival	positive			[38]
*Rana temporaria*	microsats				tadpole growth rate tadpole survival	[40]
*Hyla arborea*	microsats	tadpole body mass tadpole length tadpole stage at 37	positive positive positive *			[59]
*Bufo calamita*	microsats	*Bd* infection in population	positive 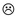		tadpole growth rate tadpole develop. time tadpole survival	[52]
*Hyla arborea*	microsats				adult body size adult body condition reproductive investment	[60]
*Lithobates sevosus*	microsats	egg survival tadpole survival	positive positive			[61]
*Anaxyrus boreas*	microsats	adult *Bd* infection	positive 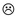			[45]
*Lithobates yavapaiensis*	microsats	adult *Bd* tolerance mortality	positive negative		infection intensity but ***GD*** lowest in uninfected pops.	[41]
*Pseudacris ornata*	microsats	adult *Bd* infection	positive 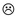			[51]
*Bombina variegata*	microsats				adult *Bd* infection	[62]
*Plethodon cinereus*	microsats	adult home range size juvenile growth rate	positive positive		juvenile survival	[63]
*Hynobius tokyensis*	microsats	egg survival	positive			[33]
*Rana ornativentris*	CytB	egg survival	positive			
*Pseudophryne cooroboree*	SNPs	adult survival after *Bd* infection	positive		adult infection load	[42]
*Rana sylvatica*	MHC II β	tadpole *Ranavirus* infection intensity	negative		*Ranavirus* infection prevalence	[64]
*Litoria verreauxii*	genomic SNPs	adult *Bd* infection	negative		adult *Bd* infection intensity	[46]
*Bombina variegata*	microsats	adult *Bd* infection	negative		adult *Bd* infection intensity	[65]
*Rana pipiens*	MHC II β exon 2 microsats/mtDNA	adult Bd infection	negative		adult *Bd* infection intensity adult B*d* infection and inf. intensity	[53]
*Eleutherodactylus coqui*	genomic SNPs	adult *Bd* infection	negative			[66]
	**Genetic locus**					
*Rana temporaria*	MHC II β exon 2	tadpole survival	allele C overrepresented, allele H underrepresented in dead tadpoles			[40]
*Bufo calamita*	MHC II β exon 2	*Bd* infection in population	MHC diversity higher in uninfected populations			[52]
*Lithobates yavapaiensis*	MHC II β exon 2	adult frog survival after *Bd* infection	MHC heterozygotes and Allele Q			[67]
*Physalaemus pustulosus*	MHC II β exon 1	*Bd* infection	*Bd* resistant allele P9 at higher frequency in pop most affected by *Bd*			[68]
*Pseudophryne corroboree*	MHC I A	*Bd* infection	some alleles pos. associated with infection load and susceptibility			[42]
*Rana sylvatica*	MHC II β	*Ranavirus* infection	intensity lowest in individuals with genotype ST1/ST7			[64]
*Atelopus varius* *Atelopus zeteki*	transcriptome sequences	survival of *Bd* infection	candidate genes related to immune system and skin integrity associated with survival			[69]
*Rana pipiens*	MHC II β exon 2	*Bd* infection	*Supertype 4* conveys increased risk of infection			[53]

* Indirect evidence, fitness increased in inter-population crosses.

### 3.3. Effects of Genetic Diversity on Ecological Fitness Traits

Tadpoles were commonly used for fitness experiments in which tadpole development or survival was measured under different ecological conditions (N = 11 studies, Table 2). Tadpoles were raised under high and low food regimes, under predation, competition or desiccation risk, different temperature treatments or exposure to a pathogen. Commonly, these studies provide evidence that fitness traits are affected by adverse conditions: in stressful situations like low food, presence of predators, desiccation risk, competition, unsuitable temperature, exposure to diseases or elevated UV light, fitness traits or survival were adversely affected. Particularly interesting are studies that found interactions between ***GD*** and treatments: four studies found evidence of fitness costs associated with low genetic variation under severe conditions [32,50,55,57]. For example, in *Rana sylvatica* mortality was higher in tadpoles of low ***GD*** when exposed to UV light [57]. Interestingly, in *B. calamita* a higher ***GD*** improved survival of tadpoles in water of cold temperatures, where survival was highest, but not in water of variable temperatures, a situation which was supposed to be stressful for the toads [38]. Overall, there is some evidence that a higher ***GD*** improves amphibian fitness under certain ecological conditions, while a low ***GD*** was never associated with better fitness. 

### 3.4. Effects of Genetic Diversity on Demographic and Health-Related Fitness Traits

In amphibians as well as in other animals, isolation and fragmentation of populations is associated with small population sizes and therefore lower ***GD***. From all studies included in this review (Table S1), thirteen found negative effects of isolation on different demographic fitness proxies (Table 3). Egg and tadpole survival was reduced in small or isolated populations in four European (*B. calamita*, Rowe and Beebee 2003; *H. arborea* [54]; *R. latastei* [55]; and *R. temporaria* [37]) one North American (*Lithobates sevosus* [61] and two Japanese species (*Hynobius tokyoensis* and *Rana ornatriventris* [33]). With respect to *Bd* infection the results are more divergent: One study on yellow-bellied toads (*B. variegata*) found that *Bd* prevalence and intensity was highest in two isolated and inbred populations [65], while in *Litoria verrauxii* from Australia *Bd* infection was absent in an isolated population [46]. Across six Brazilian species *Bd* infection was more intense in fragmented populations as compared to non-fragmented populations [34].

## 4. Discussion

Our review analysed the relationships among ***GD*** and fitness traits, in some cases together with population size and fragmentation, in amphibian populations. Most studies did not contain information on all of these items, and the number of species investigated is extremely small (N = 20 species) when considering the 8689 known amphibian species [70]. Even with these limitations, however, the overall picture is that declines in amphibian population sizes are associated with a decrease in ***GD***, with a concomitant negative effect on fitness. This is in accordance with earlier reviews on GFC in amphibians, pointing out the relationship between small population size, genetic diversity depletion or inbreeding, and the unfavourable consequences on survival [29,71]. The state of this research field is, however, far from satisfactory: Future studies need to include a more diverse assemblage of species—for example, more species from the unstudied but highly diversified tropical families in Anura, evolutionarily distinct lineages within Caudata and Gymnophiona, genetically diverse hybridogenic species [72,73], and species using different reproductive modes and parental care strategies such as aquatic oviparity or terrestrial direct development [74,75]. Additionally, studies that link the measured fitness traits with the lifetime survival and reproductive success of populations are desirable. 

### 4.1. Genetic–Fitness Correlations

In this report, it became apparent that ***GD***, mostly measured at microsatellite loci, is often positively correlated with fitness traits. Fitness traits were mostly measured as traits that are related to the development of eggs and tadpoles or in some cases as *Bd* infection. The fitness traits of eggs and tadpoles (e.g., survival and growth rate) are generally positively correlated with increasing ***GD*** meaning that individuals in populations with a higher ***GD*** are more likely to survive and reach adulthood. 

However, the situation is different for the infection with *Bd*. We considered *Bd* infection an adverse fitness trait since *Bd* infection has led to disease outbreaks, mass mortality and population declines. Even though the fungus is widely distributed—it has been detected in 86 of 119 countries (72%) and in 1062 of 1966 tested species (54%; [76])—the most severe declines have occurred in Meso- and South America, whereas the situation is somewhat better in Europe, North America and Africa, and no declines have been reported from Asia [23]. In our review, we found four studies in the Neotropics including nine species, which reflects this pattern of severity.

Interestingly we found three studies that documented *Bd* infection to be positively correlated with ***GD*** [45,51,52]. One possibility to explain this paradox, if we assume that a higher ***GD*** is coupled with better health conditions, is that ***GD*** is higher in those populations that are more connected with others via migration and gene flow. While contact with migrating individuals increases genetic exchange and therefore ***GD***, it also increases the exposure to parasites and pathogens [45]. By the same logic, the fungus might not spread into isolated amphibian populations with less ***GD,*** which might explain the observed correlations. In contrast, some other studies show a negative relationship between ***GD*** at different genetic markers and *Bd* infection. One example is *L. verreauxii* [46], in which frogs with a higher H_O_ were less likely to be infected; a similar result was found for *B. variegata* [65]. Those studies highlight the importance of individual ***GD*** for resisting the infection in populations connected by gene flow. We found no study that related ***GD*** to the infection with *Batrachochytrium salamandrivorans* (*Bsal*). The host specificity of this fungal parasite is largely restricted to Caudata [77], and associations between ***GD*** and *Bsal* infection appear to have not been studied or published to date.

Even though many studies showed positive relationships between ***GD*** and fitness proxies, while only a few studies showed negative correlations, nearly 46% of the calculated statistical tests were not significant (Figure 3). Even within the same species, some fitness proxies were positively correlated with ***GD*** while others were not. For example, in *B. calamita,* tadpole growth rate was positively correlated with ***GD*** and egg hatch rate was lower in genetically depleted populations, but tadpole survival was not affected by ***GD* [49]** and was better in a large population with higher genetic diversity compared to a small population in another study [48]. This observation points to a problem: for most species it is not clear which fitness proxies are actually related to fitness in the sense of long-term survival and reproduction. Therefore, it would be preferable to always measure a set of fitness proxies, and ideally this would be performed in several localities covering distinct environmental conditions.

### 4.2. Population Size and Fragmentation

Conservation of ***GD*** is not only important for amphibians. A comparison between threatened and closely related non-threatened animal and plant species revealed that the genetic diversity or heterozygosity is on average 35% reduced in the threatened group. Seemingly, inbreeding depression reduces the reproductive fitness and therefore increases extinction risk and lowers the evolutionary potential of depleted populations [78]. Conservation of ***GD*** is therefore of immense importance in small populations which otherwise are put at a high risk of extinction. At this point, we want to emphasize that in large and outbred amphibian populations, fitness traits have not been found to be correlated with ***GD***, e.g., [32]. Instead, the negative effects of low ***GD*** seem to come into play when genetic depletion is significant and especially when genetic load and inbreeding are present [32,61]. 

In most studies reviewed for this report, inbreeding coefficients and estimates for population sizes are not calculated or presented. Nevertheless, we found substantial evidence for the positive association between connectivity and population size with ***GFCs***. In eleven studies, egg and tadpole survival and development were reduced or *Bd* infection was more severe in isolated and/or small populations (Table 3). Notably, inbred tadpoles of *L. sevosus* had a lower probability of survival in an isolated population. The consequence of the elevated mortality of inbred tadpoles is the reduction in the inbreeding coefficient in later stages (juveniles and adults) because only outbred, genetically more diverse individuals survive the tadpole stage. This case emphasizes the need to measure amphibian inbreeding at different developmental stages [61]. 

### 4.3. Candidate Loci and Adaptive Markers

Some studies highlight the importance of investigating the role of adaptive markers for amphibian fitness. Those studies have been conducted mainly in the context of *Bd* infection (Table 1). Besides the importance of diversity at the MHC loci, certain alleles (e.g., allele Q and P9; [67,68]) seem to convey resistance and survival. Overall, the knowledge on the interaction between amphibian fitness and adaptive loci is minimal, and we hope to find more outcomes from this type of research in the future. 

### 4.4. Interactions with Environmental Conditions

Several studies included varying environmental factors (food, predation, competition, desiccation, temperature, pathogens and sun exposure) together with ***GD*** in their experimental design (Table 2). Four of those studies found an interaction between ***GD*** and one ecological factor. In three studies the survival of tadpoles under stressful treatment was better when ***GD*** was higher [50,55,57], while ***GD*** was correlated with survival in the benign treatment in another study [38]. Even though the number of studies is low, they underline how low ***GD*** may reduce the ecological fitness of amphibians. 

### 4.5. Recommendation for Further Studies

One problem detected in this research is the lack of standardisation of methodologies. Even though many studies investigated the effect of isolation and/or low ***GD*** on tadpole fitness, and ***GD*** was mostly evaluated with microsatellites, most of them varied in the fitness proxies that were measured (Table 1). Moreover, it is mostly unknown how fitness traits measured at different developmental or life stages are correlated with each other. While ecological conditions (e.g., food limitation [79]) affect the mass and developmental time to metamorphosis, several studies found a positive relationship between size at metamorphosis and later survival [80]. To the contrary, a long-term study including demographic modelling found that lifetime fitness of spadefoot toads in Austria (*Pelobates fuscus fuscus*) was weakly affected by variations in size and timing at metamorphosis [81]. These uncertainties need more research attention in the future. In most cases, measuring the relationship of fitness across life stages will remain impractical or even impossible with current funding schemes. We therefore suggest (nearly) non-invasive methods: sampling the tail tips of tadpoles and the saliva of juvenile and adult amphibians for molecular methods. Furthermore, we recommend reporting the level of isolation of the study localities, reporting ***GD*** as population H_O_, H_E_ and individual MLH, and providing at least a rough estimate of population size or effective population size (N_E_). Currently, the use of microsatellites is declining and is increasingly being replaced by more informative methods like SNPs, where the number of molecular markers is increased a thousandfold (e.g., [82]). The effects of other adverse factors such as agrochemicals, direct exploitation and invasive species [83] on amphibian ***GD*** and fitness is another research gap. Identifying, monitoring and measuring harmful ecological conditions together with ***GD*** and population sizes of endangered species is a process of decades. To be successful it needs long-term funding and stable personnel conditions in species protection centres and scientific institutions. 

A matter of current and future interest should be the correlation between neutral genetic variation and adaptive variation as well as the presence of adaptive loci that help to counteract particular adverse environmental conditions. For example, only a few studies to date have found adaptive loci associated with climatic conditions [82,84,85,86] despite the evident risk climate change poses on amphibians. 

## 5. Conclusions

Multiple studies have shown the importance of keeping ***GD*** above critical levels for maintaining healthy and fit populations [78,87]. Low ***GD*** in small populations not only lowers the fitness of the animals but it also compromises the adaptive potential, e.g., the ability to cope with environmental changes, by modifying allele frequencies of critical genes that might prove beneficial under future conditions. In too small populations, these critical genes are at risk of being lost by chance, i.e., by genetic drift, and the probability that the genetic variants are locally adapted to current local conditions is also low. Because of these genetic inconveniencies, amphibian species protection measures should aim to preserve and manage meta-populations—subpopulations connected by gene flow—with an effective populations size (N_E_) of more than a thousand individuals [88]. One problem is that the N_E_ are often unknown and hard to infer from the population size, N, in amphibians, but fortunately they can be estimated from genetic markers [89]. In amphibians, those meta-populations consist of subpopulations tied to certain resources such as breeding ponds among which migration and gene flow take place. Small and disconnected populations with low ***GD*** facing extinction risk should be reconnected to larger, geographically close meta-populations via steppingstones and artificial crossing aids such as ditches, tunnels or green bridges. If this is not possible, genetic rescue by the introduction of individuals from larger populations that are genetically diverse or by mixing of individuals from several source populations for translocation [16,90] could serve to enhance ***GD***. In the latter case, the risk of outbreeding depression can be avoided by applying some simple rules developed by experts [91] and by limiting the introduced fraction to 5–20% of the recipient population. A review by Frankham [17] on genetic rescue revealed that the introduction of individuals from other populations into small plant, invertebrate and vertebrate populations generally resulted in higher fitness. The author concluded that the method of genetic rescue should be used more often in species protection measures.

With respect to changing environmental conditions caused by climate change or the spread of novel or introduced pathogens, it is important to unravel those critical genes that provide resistance to new negative influences (e.g., greater drought) by applying genomic methods. Once those genes have been identified, beneficial variants should be introduced into the affected threatened populations. For example, in the Yosemite toad, *Anaxyrus canorus*, 24 candidate genes have been identified that seem to be under climate-related selection [85]. Identifying more critical genes under selection by environmental change and how they might contribute to species conservation, together with the maintenance of a substantial level of local genetic variation, is the next challenge in genetic amphibian conservation science. This kind of research will aid the efforts of upcoming international conventions aiming to restore and maintain ***GD*** within and among populations of all species (global biodiversity framework, GBF, [92]).

## Figures and Tables

**Table 2:** Summary of studies that experimentally evaluated the effect of ecological factors on fitness traits in amphibians. ***GD*** = genetic diversity, *Bd* = *Batrachochytrium dendrobatidis* (chytrid fungus). **IA** = interaction with the ***GD*** in the population.

Species	Treatment	Fitness Trait	Result	Citation
*Bufo calamita*	food: low vs. high	tadpole survival tadpole growth rate tadpole develop. rate tadpole time to metamorphosis	All fitness traits were better under a high food regime.	[32]
*Rana temporaria*	food: low vs. high	tadpole survival tadpole growth rate tadpole develop. rate tadpole time to metamorphosis	All fitness traits were better under a high food regime.	[32]
*Bufo calamita*	predation: yes or no competition: yes or no desiccation: yes or no	tadpole survival tadpole growth rate	Reduced by predation and desiccation; **IA** with pop.; reduced by predation and competition; **IA** with pop.	[48]
*Rana temporaria*	temperature: 14 °C, 18 °C and 21 °C food: low vs. high	tadpole survival	**IA**: in stressful treatment (e.g., low food or cold temp.), survival was better at higher ***GD*** and less-related parents.	[50]
*Rana latastei*	exposure to *Ranavirus*: low vs. high	tadpole survival	Reduced by high exposure. **IA**: survival was better when ***GD*** was higher.	[55]
*Rana sylvatica*	UV-B light: sunlight, filter and acetate sheet	tadpole survival tadpole deformity	Lowest under direct sunlight. **IA** between ***GD*** and UV light was highest under direct sunlight.	[57]
*Bufo calamita*	temperature: 19 °C, 27 °C and variable	tadpole survival	Highest under cold treatment. **IA** with ***GD***.	[38]
*Rana temporaria*	temperature: 10 °C, 14 °C and 21 °C	tadpole growth rate	Higher in medium and high treatment.	[40]
*Lithobates yavapaiensis*	*Bd*: yes or no	adult frog survival	Uninfected individuals survived better.	[67]
*Hyla arborea*	*Bd*: low vs. high dose	tadpole time to metamorphosis tadpole mass survival of froglets	Longer time at high *Bd*; mass reduced at high *Bd*; treatment n.s.	[47]

**Table 3:** The effect of isolation and/or small population size on fitness proxies in amphibian species.

Species	Negative Effect of Isolation or Small Population Size	Citation
*Bufo bufo*	Survival was higher and deformity was lower in larger, less isolated populations.	[36]
*Bufo calamita*	Low hatch rate in the smallest isolated populations.	[49]
*Hyla arborea*	Tadpole survival was lower in isolated ponds.	[54]
*Bufo calamita*	Tadpole survival and growth rate were lower in small populations.	[48]
*Rana latastei*	Tadpole survival was reduced in isolated populations.	[55]
*Rana latastei*	Egg hatch rate was reduced in isolated populations.	[39]
*Rana temporaria*	Tadpole body size and survival was reduced in the fragmented compared to the continuous habitat.	[37]
*Hyla arborea*	Individual performances were reduced in isolated, small populations compared to large, non-fragmented populations.	[59]
*Hyla arborea*	Stronger high *Bd* dose effect on fitness traits (Table 2) in isolated populations.	[47]
*Lithobates sevosus*	Inbred tadpoles did not survive in isolated population.	[61]
*Hynobius tokyoensis* *Rana ornativentris*	Genetic diversity and egg survival were positively affected by the forested area.	[33]
*Bombina variegata*	*Bd* prevalence and *Bd* intensity were highest in two inbred, isolated populations.	[65]
*Litoria verreauxii*	No *Bd* infection in isolated populations.	[46]
*Six tropical species* (no data for single species)	*Bd* infection was higher in fragmented populations relative to continuous populations.	[34]

## Data Availability

Data sharing is not applicable.

## Supplementary Materials

The following supporting information can be downloaded at: https://www.mdpi.com/article/10.3390/ani13223564/s1. Table S1 contains detailed information on the species studied, molecular markers, genetic diversity measures, experiments and genetic–fitness correlation as well as the main conclusions from the studies included in this review.
