# Supplementary material for: Importance of Genetic–Fitness Correlations for the Conservation of Amphibians
Source: Animals (Basel). 2023 Nov 18;13(22):3564. doi: 10.3390/ani13223564 (PMC10668650; doi:10.3390/ani13223564)
Supplement: Supplementary file 1 [file animals-13-03564-s001.zip › animals-2681210-supplementary.pdf]

Table S1: Information summary on species and their genetic diversity (***GD***), fitness proxies and correlations between both as well as interpretation of the results

Species, study country, region, no. and size of SS	Molecular marker / method	Genetic diversity	Proxy for fitness	Statistical model	Correlation<br>**Bold = significant**	Remarks / Interpretation of results	Citation
Bufo bufo<br>UK, TE<br>12 SS: 1) 8 small urban pop, N = 6 - 200 adults;<br>2) 4 large rural pop, N = 500 – 5000 adults	27 allozymes<br>(12 SS)<br><br><br>3 minisats<br>(5 SS)	Urban Rural<br>F_{ST} = 0.53 0.29<br>P = 14.8% 25%<br>Na/l= 1.18 1.36<br>F_{ST} = 0.23 0.066<br>H_O = 0.035 0.017<br>Na/l= 3.89 5.00<br>H_O = 0.36 0.43	Tad survival<br>Tad deformity	Correlation analyses	Survival Deformity<br>P: **r = 0.74**<br>Na/l: r= **0.67**<br>H_O :r = -**0.65**<br>Na/l: r= 0.85	Higher ***GD*** in large rural pops than in smaller urban pops. ***GD*** is pos. correlated with tadpole survival, but neg. with developmental abnormalities; reduction in ***GD*** and fitness in urban toads	Hitching & Beebee 1998 [36]
Bufo calamita<br>UK, TE<br>38 SS (34 in UK)<br>variable N	8 microsats<br>33 SS	P = 38-75%<br>Na/l = 1.5 – 2.8<br>H_E = 0.19-0.34<br>H_O = 0.22-0.38	Tad survival<br>Tad growth rate<br>In 6 test SS	Correlation analyses	No correlation between survival and ***GD***; growth rate was correlated with H_E (**r**=0.6 **- 0.9**, P< 0.01), H_O, P, Na/l; low hatch rate in pop with lowest ***GD***	H_E lower in range edge pops; H_E pos. correlated with fitness (growth rate); tad from smallest and isolated pop exhibited lowest fitness and H_E	Rowe et al. 1999 [49]
Hyla arborea<br>Sweden, TE<br>10 SS (ponds)	18 allozymes	Mean ***GD***: 1.06 alleles per locus	Egg hatch rate<br>Tadpole survival	Correlation analyses	Very low ***GD***; no correlation with fitness	Survival was lower in isolated ponds compared to more central ponds	Edenham et al. 2000 [54]
Bufo calamita<br>UK, TE<br>1 SS: large natural population, N ~ several thousand adults	5 microsats<br>Experiment: high & low food regime	For individual tadpoles:<br>H = 0.42 – 0.73<br>Mean d^2 = 5.76-14.02<br>Outbreeding d^2 = 12.2 – 25.5<br>Scaled d^2 = 0.13 – 0.19	Tad survival<br>Tad growth rate<br>Tad developm. rate<br>Tad time to metamor.	Correlation analyses	No correlation between fitness and genetic measures; but fitness better under high food condition	No relationship between fitness related traits and individual genetic diversity	Rowe & Beebee 2001 [32]
Rana temporaria<br>UK, TE<br>1SS: large natural population with immigration, N ~ tens of adults	7 microsats<br>Experiment: high & low food regime	For individual tadpoles:<br>H = 0.50 – 0.65<br>Mean d^2 = 26.5 – 62.4<br>Outbreeding d^2 = 36.1 – 134.0<br>Scaled d^2 = 0.01 – 0.067	Tad survival<br>Tad growth rate<br>Tad develop. rate<br>Tad time to metamor.	Correlation analyses	No correlation between fitness and genetic measures¸ but fitness better under high food condition	No relationship between fitness related traits and individual genetic diversity	Rowe & Beebee 2001 [32]
Bufo calamita<br>UK, TE<br>2 SS: 1) large pop several hundred toads	8 microsats<br>Experiment: Pop X predation X competition X	No data: lower ***GD*** in small compared to large pop (extracted from Rowe et al. 1999)	Tad survival<br>Tad growth rate	Correlation analyses	Survival and growth rate (during first 10 days) higher in large than small pop. Predation & desiccation reduced survival; predation	Lower fitness in small than large pop; best explained by increased genetic load in small isolated pop; pop at risk due to small N_E	Rowe & Beebee 2003 [48]

2) small isolated pop<br>$N_C < 50$	desiccation treatment				& competition reduced growth rate		
Rana temporaria<br>Finland, TE<br>4 SS<br>In all pops females N < 100	8 microsats<br>Experiment:<br>3 temp. treatm:<br>14°C, 18°C, 22°C<br>Low & high food	Individual Measures:<br>Parents: Ho, d^2, rxy<br>Tad: H_{EST} and d^2_{EST} for tadpoles	Tad survival<br>Tad develop. rate<br>Tad growth rate	GLMM	Survival neg. correlated with r_{xy}, pos. correlated with H_{EST}, (d^2_{EST} n.s.), develop. rate pos. correlated to d^2_{EST}, growth rate neg. correlated with d^2_{EST}	***GD*** interacts with food and temperature; **GFCs** are present for survival, seem to be sensitive to environment, more pro-nounced in stressful condition	Lesbarrères et al. 2005 [50]
Rana latastei<br>Italy, Slovenia, TE<br>6 SS: embryos from 10 clutches per pop	6 microsats<br>Experiment: high and low expo-sure of tad to *Ranavirus*	AR<br>HS<br>P	Tad survival	GEE, Regression analyses	Better survival in population with higher ***GD*** in low exposure treatment (P>0.0001); high exposure led to high mortality	Lower survival in western isolated pops with low ***GD*** when exposed to novel pathogen; eastern pops have higher potential to evolve resistance	Pearman & Garner 2005 [55]
Rana sylvatica<br>Connecticut, TE<br>1 SS: N ~ 110 adults, 292 metamorphs (+ 1 SS with too low sample size)	9 microsats	Parental r_{xy}<br>Eggs / Tad: MLH	<u>In the wild</u><br>Sibship survival to metamorphosis<br>Indiv. tadpole weight<br><u>In the lab</u><br>Egg survival<br>Tadpole survival<br>Tadpole weight<br>Tadpole devel.stage	Linear regression	Sibship survival pos. correlated with MLH (**R^2 = 0.30**) and neg. correlated with r_{xy} (**R^2 = 0.29**), weight not correlated with ***GD***<br><br>no correlation of fitness proxies with MLH or r_{xy} in the lab	Inbreeding (measured as MLH or r_{xy}) negatively affects survival in the wild, but not in the lab	Halverson et al. 2006 [56]
Rana sylvatica<br>Ohio, TE<br>12 SS	RAPD<br>Experiment: 3 UV-B radiation treatm. of eggs: sun light, filter, acetat sheet	GSI for each population (no data)	Egg survival<br>Tad survival<br>Tad deformity	MANOVA	All 3 fitness traits neg. correlated with ***GD***; Low ***GD*** correlated with higher larval mortality and deformity when exposed to UV-B light (both P > 0.0001)	Fragmented populations with low ***GD*** might be at increased risk of mortality when interacting with environmental stressors like direct sun radiation	Weyrauch & Grubb 2006 [57]
Rana latastei<br>Italy, TE<br>10 SS: 4 isolated, 6 non-isolated pops	6 microsats	Non-isolated / Isolated<br>Mean pw F_{ST} = 0.18 /F_{ST} = 0.34<br>Estimate of ***GD***: PCA score including AR, P, H_O<br>AR = 1.4 – 2.6<br>H_O = 0.12 – 0.23<br>H_E = 0.16 – 0.24	Egg hatch rate	Correlation analyses	No correlation between ***GD*** and hatch rate (r=0.14, P=0.75), correlation (**r=0.82**, P=0.023) when controlling for distance from glacial refugium; Hatch rate higher in continuous (0.77-0.98) than isolated (0.38-0.54) pops.	Overall ***GD*** is low; ***GD*** lost through recent isolation had negative effect on fitness; habitat restoration is recommended	Ficetola et al. 2007 [39]

Rana temporaria<br>Sweden, TE<br>6 areas with 9 SS each;<br>3 fragmented (F) and<br>3 continuous (C)<br>areas: N_F= 6-154, N_C= 25-1470	7 microsats<br>Experiment:<br>4 fragmented &<br>4 continuous SS<br>with low vs. high<br>***GD***	Fragmented / Continuous<br>Global F_{ST} = 0.071 F_{ST} = 0.02<br>AR = 3.3 – 3.7 AR = 4.3 – 5.0<br>H_O = 0.50-0.62 H_O = 0.71-0.78<br>HS = ??	Tad body size<br>Tad survival probab.	Correlation analyses	Body size Survival probab.<br>Ho: **r =0.73** Ho: **r = 0.77**<br>Hs: r = 0.70 Hs: **r = 0.88**<br>AR: r = 0.31 AR: r =0.70	Higher ***GD*** in continuous habitat; positively correlated with fitness traits; larvae from frag. habitat were smaller and survived less	Johansson et al. 2007 [37]
Rana temporaria<br>Finland, TE,<br>2 SS = 2 ponds,<br>small N	8 microsats	Individual H_O of tad, father(sire) and dam (mother)	Tad weight<br>Tad age at metamorphosis	GLMM	Tad weight was influenced by H_O (Linear Model; P<0.001) and dam H_O (P<0.047); no genetic but "pond" influence on age at metamorph.	Since weight is correlated with fitness in amphibians, ***GD*** might be an important component of individual fitness *in R. temporaria*	Lesbarrères et al. 2007 [58]
Bufo calamita<br>Sweden, TE<br>6 SS<br>all pops are large	105 AFLPs<br>Experiment: 3 temp. treatm: 27°,19°C,variable	Global F_{ST} = 0.16<br>Pairwise F_{ST} = 0.06-0.27<br>47-91% variable AFLP loci	Tad survival	GLMM	***GD*** positively correlated with survival in cold treatment (19°C) where survival was highest (P = 0.016)	Low genetic variation does not seem to cause a fitness cost under stressful condition (variable temp.)	Rogell et al. 2010 [38]
Rana temporaria<br>UK, TE<br>8 SS<br>Urban (4 SS) and rural (4SS) habitats	9 microsats<br>MHC II β exon 2<br>Experiment:<br>3 temperature regimes: 10°C, 14°C, 21°C	Mean pw Msat F_{ST} = 0.051,<br>Mean pw MHC F_{ST} = 0.099<br>Microsats: Rural Urban<br>AR = 6.3-6.9, AR = 5.8-6.8<br>H_O =0.62-0.74 H_O = 0.68-0.84<br>H_E =0.68-0.72, H_E = 0.68-0.73	Tad growth rate<br>Tad survival	Correlation analyses,<br>Chi^2-Test	No correlation between ***GD*** (both markers) and fitness; Dying vs. surviving tad: MHC allele *C* was over-represented, allele *H* was underrepresented in dead tad	No differences in ***GD*** or survivorship between rural and urban pops; growth rate higher in urban areas. Functional loci are important when assessing pop genetic health	Zeisset & Beebee 2010 [40]
Hyla arborea<br>France, TE<br>4 SS: 2 large and 2 small, isolated & genetically eroded pops	15 microsats<br>Experiment:<br>inter- and intrapopulational crosses	Estimates of H_{EST} per family	Tad body mass,<br>Tad length<br>Tad stage at day 23	Linear model	Tad performances better in non-frag. pops than isolated pops; correlated with H_{EST}:<br>Stage: F = 14.19, P < 0.001;<br>Mass: F = 9.18, P = 0.003;<br>Length: F = 10.77, P = 0.001<br>Stage and length increased in interpop. crosses in isolated pops; mass increased in crosses from isolated and non-frag. pops	Inbreeding depression (within) vs fixation load (among pops)? Support for genetic drift leading to fixation of slightly deleterious alleles; translocating individ. from moderately divergent pops might increase fitness in tadpole stage	Luquet et al. 2011 [59]
Bufo calamita<br>UK, TE<br>Region 1: uninfected,<br>N_C = 140	8 microsats<br>MHC II β exon 2	<u>Microsats</u>:<br>Region 1 Region 2<br>H_E = 0.1 H_E = 0.41<br>AR = 1.33 AR = 3.14<br>5 <u>**MHC**</u> allele frequency	Tad growth rate<br>Tad develop. time<br>Tad survival	KW-ANOVA,<br>Chi^2-Test	Microsat ***GD*** not correlated to fitness. No differences in fitness between infected and uninfected region; toads in both areas equally healthy.	Certain MHC alleles might be associated with infection. Directional selection favouring alleles conferring resistance to ***Bd***	May et al. 2011 [52]

Region 2: infected with ***Bd***, N_C = 282					MHC diversity higher in uninfected region.		
Lithobates yavapaiensis<br>Arizona, ST<br>5 SS	MHC II β exon 2<br>14 microsats<br>***Bd*** infection experiments: yes or no	Individual and population MHC heterozygosity and MHC allele frequencies<br>Population structure (microsats): K = 6	Adult frog survival	Cox proportional hazard model, Fishers exact test	MHC heterozygotes & MHC allele Q associated with survival	Variation among pops in *Bd* infection and MHC alleles. Fitness advantage conveyed by MHC heterozygosity & allele Q	Savage & Zamudio 2011 [67]
Hyla arborea<br>France, TE<br>5SS: 3 large and 2 small, isolated & genetically eroded pops	Intrapopul. Crosses: 5 – 9 clutches<br>Low & high ***Bd*** dose	***GD*** known from previous studies	Tad stage at metamorphosis<br>Tad mass<br>Tad time to metamor.<br>Survival of froglets	GLMM, ANOVA, ANCOVA	No ***Bd*** detection in adults or tad; Longer time to metamor. in isolated pops exposed to high ***Bd***; Mass higher in isolated pops, but more reduced when exposed to high ***Bd***	Tad seem to prevent or clear infections rapidly;<br>At high ***Bd*** dose survival of froglets from nonfrag-mented pops. slightly higher, but survival higher & higher mass of isolated tadpoles in control group	Luquet et al. 2012 [47]
Hyla arborea<br>France, TE<br>4 SS: 2 large and 2 small, isolated & genetically eroded pops	15 microsats<br>n = 150 females, 296 males	Na/l = 3.6 – 5.6<br>AR = 3.6 – 5.5<br>H_E = 0.37-0.46<br>H_O = 0.38 -0.46<br>F_{IS} = -0.08 – 0.04<br>Indiv. MLH	Adult body size<br>Body condition<br>Reproductive investment: males: chorus attendance; females: clutch mass & egg size	Linear models	No correlation between fitness measurements and indiv. MLH; larger frogs invested more in reproduction.	Lack of **GFC** on adult traits probably because inbreeding is low in these populations; (*inbreeding avoidance by female mate choice*?); or selection against unfit genotypes in tad	Luquet et al. 2013 [60]
Lithobates sevosus<br>Mississippi, ST<br>1 SS, isolated and small pop size, probably inbred and bottelnecked	8 microsats	Individuals: eggs metamorphs, adults:<br>MLH<br>IR	Egg mortality<br>Tad survival to metamorphosis	Regression analyses	MLH positively correlated with survival of egg clutches (**r^2 = 0.15**);<br>Metamorphs had lower F_{IS}, lower IR and greater MLH than adults and eggs!	Conclusion: most inbred tad did not survive to metamorphosis; survival of individual with greater ***GD*** might prolong persistence of isolated pop	Richter & Nunziata 2013 [61]
Anaxyrus boreas<br>USA, Montana, TE<br>One large sample area:<br>Glacier National Park	11 microsats<br>***Bd***: n =199	AR = 9.9-13.0 (for 3 groups)<br>H_O = 0.62-0.71<br>H_E = 0.68-0.74<br>F_{IS} = 0.04-0.09<br>Indiv. MLH<br>Population structure: K=2	Adults (> 1year old)<br>***Bd*** presence	Hierarchical logistic regression model	Overall toads have high genetic diversity<br><br>MLH was positively related to ***Bd*** infection (P=0.03)	Population effect: immigration might increase heterozygosity as well as greater exposure to ***Bd***	Addis et al. 2015 [45]
Lithobates yavapaiensis<br>Arizona, ST<br>12 SS	14 microsats<br><br>19 bioclimatic variables	pw F_{ST} = 0.17-0.60 (mean = 0.32)<br>AR, $F_{IS,}$<br>H_O = 0.38 – 0.68	Adults<br>***Bd*** intensity<br>***Bd*** prevalence	GLMs	AR + H_O highest in ***Bd*** tolerant pops, intermediate in susceptible, and lowest in uninfected pops; Host ***GD***	Warmer temp. associated with higher ***Bd*** prevalence & intensity; Mortality lower in pops with higher	Savage et al. 2015 [41]

	(related to temp. & precipitation)	Population structure: K=10	Mortality prevalence per population		(PC calculated from Ho, Fis, AR) alone explained mortality **r^2 = 0.499**, P=0.015	***GD*** measures; ***GD*** lowers ***Bd*** susceptibility, important to preserve ***GD*** for species persistence	
Physalaemus pustulosus, Panama, TR<br>3 SS: 2 lowland, 1 highland, sampled 2010 & 2103	5 microsats<br>MHC II β 1	High genetic diversity:<br>H_E = 0.57 - 0.93 (across loci)<br>MHC II β 1 allele frequency	Adults<br>***Bd*** infection	?	Lowland frogs less infected than highland frogs; higher frequency of MHC II β1 P9 alleles associated with resistance to ***Bd*** in highland populations	Strong selection on MHC II β1 P9 alleles in highly infected populations. Potential fitness cost: Homozygosity at this loci may decrease capacity to bind antigens of other pathogens	Kosch et al. 2016 [68]
Pseudacris ornata SE USA, ST<br>15 SS	7 microsats<br>n = 327	AR<br>H_E	Adults<br>***Bd*** infection<br>11 (out of 15) pops. infected with Bd	GLMs	Pop average H_E correlated with ***Bd*** prevalence; Decreasing temperature correlated with increasing ***Bd*** prevalence and ***Bd*** intensity	Possibly in pop. with now low prevalence, selection has pushed tolerant genotypes towards fixation resulting in decreased heterozygosity	Horner et al. 2017 [51]
Bombina variegata Germany, TE<br>19 SS<br>N_E = 24-128	6 microsats<br>n = 274	Indiv. MLH<br>H_E	Adults<br>***Bd*** infection<br>14 out of 19 pops infected with ***Bd***	GLMs	No correlation between indiv. MLH or pop level H_E with ***Bd*** infection	***Bd*** presence did not influence survival or body condition; no evidence that genetic diversity or environmental variables affect ***Bd*** infection probability	Wagner et al. 2017 [62]
Plethodon cinereus Virginia, TE<br>1SS	7 microsats<br>n = 109 adults<br>n = 35 juveniles	Indiv. MLH	Adult home range size<br>Juvenile growth	GLM, GLMM	MLH pos. correlated with home range size; tendency that MLH pos. affects growth, no effect of MLH on survival	***GD*** might influence behaviour and growth via foraging	Liebgold et al. 2018 [63]
Hynobius tokyoensis Japan, ST<br>32 SS<br>N_C = 1 -117	Cytb<br><br>5 microsats	π = 0.00 – 0.04<br><br>H_E = 0.10 – 0.51	Hatchability of clutches in the field	Path analysis	For both species: Temperature, altitude & land-use explained ***GD***		Okamiya & Kusano 2018 [33]
Rana ornativentris Japan, ST<br>38 SS<br>N_C = 2 - 500	Cytb<br>4 climatic, 3 topographic & 4 land-use variab.	π = 0.00-0.24	Hatchability of clutches in the field	Path analysis	***GD*** positively affected mean hatchability (path analysis coefficient = 0.27, for both species together)	Forested area ratio positively affected population size (n.s.), ***GD*** and mean hatchability	

Pseudophryne corroboree<br>Australia, ST<br>4 SS, small census size<br>N_C = 13 males	SNPs<br>n = 16 - 22<br><br>MHC IA<br>n = 16 – 22<br>Experiment: ***Bd*** infection	Indiv. genome-wide H<br>H_E = 0.33 – 0.37<br>H_O= 0.36 – 0.40<br>F_{IS}= -0.050 – -0.073<br>AR= 1.34 – 1.38<br>F_{ST} = 0.106 – 0.191<br>Population structure K=2	Adult frogs<br>Infection load<br>Number of days survived	GWAS	Indiv. genome-wide H pos. correlated with survival;<br>1 MHC allele associated with high infection load; 2 MHC alleles more common in susceptible pops, one MHC allele neg. associated with survival; some putative adaptive SNPs associated with survival (n.s.)	***GD*** associated with survival; the resistant pop M had highest level of ***GD***; Adaptive SNP and MHC variants important for breeding more resistant individuals.	Kosch et al. 2019 [42]
Rana sylvatica<br>Maryland, TE<br>15 SS (ponds)	MHC IIB exon sequencing and supertyping<br>n = 381	π = 0.059<br>T*D* = 1.72	Tad *Ranavirus* prevalence<br>*Ranavirus* infection intensity	Hurdle model	Infection intensity was associated with MHC heterozygosity (P<0.001), lowest in indiv. with MHC genotype ST1/ST7; prevalence without genetic association	MHC heterozygotes have a 23fold lower infection intensity compared to homozygotes; MHC functional genetic variation is important for *Ranavirus* suceptibility	Savage et al. 2019 [64]
Litoria verreauxii<br>Australia, alpine, ST<br>10 SS:<br>2 with & 8 without ***Bd***	Genomic DNA:<br>SNPs (GBS)<br>6 SS<br>n = 50 each site	Indiv. H_O<br>mean number of alleles: 1.37-1.91<br>mean H_O = 0.12-0.26,<br>mean H_E = 0.11-0.23<br>Population structure: K=10	Adult frogs<br>***Bd*** infection presence<br>***Bd*** infection intensity	GLMs, Fishers exact test	No differences in ***GD*** between sample sites with and without ***Bd***; No relationship between ind. infection intensity and H_O; but frogs with higher H_O were less likely to be infected	Large pop effect on infection rate; No ***Bd*** in isolated pops;<br>Maintenance of genetic diversity is crucial in pops infected with ***Bd***	Banks et al. 2020 [46]
Bombina variegata<br>Germany, TE<br>16 SS (7 focal sample sites, including 2 inbred subpops)	9 microsats<br>n =300<br>***Bd*** infection<br>n =577	Indiv. MLH<br>H_E = 0.49-0.65,<br>H_O = 0.34-0.73<br>HS = 0.44-0.55<br>F_{IS} = -0.4 – 0.26<br>AR = 1.48-1.59<br>Npa = 0-6<br>Population structure: K = 3	Adult toads<br>***Bd*** prevalence per pop<br>***Bd*** presence / indiv.<br>***Bd*** intensity/ indiv.<br>***Bd*** in 15 out of 16 SS<br>Prevalence: 7.1 - 85.7%<br>Intensity: 0 - 570 GE	GLMs	Infection presence correlated with indiv. MLH;<br>No correlation between MLH and infection intensity	No ***Bd*** associated mortality;<br>***Bd*** infection prob. highest in toads with low genetic diversity and isolated, inbred pops.	Oswald et al. 2021 [65]
Rana pipiens<br>Northamerica, TE<br>14 SS<br>N_E = 37 - 4861	7 microsats<br>n = 428<br>mtDNA haplotypes<br>n = 94	H_E = 0.59 – 0.92<br>H_O = 0.67 – 0.89<br>AR = 5.5 – 13.9<br>H_E = 0.00 – 0.76<br>T*D* = -2.03 – 0.77<br>H_E = 0.00 – 0.90	Adult frogs<br>***Bd*** prevalence<br>***Bd*** intensity<br>***Bd*** in 8 out of 14 SS<br>Prevalence : 5.0 – 100%	GLMs	***Bd*** prevalence neg. correlated with MHC H_E<br>MHC allele *Rapi*03* associated with reduced risk of infection (n.s); MHC *supertype 4* associated with	Populations (2SS) with smallest N_E also had lowest MHC diversity; MHC ***GD*** correlated with prevalence; neutral ***GD*** not correlated with ***Bd***.	Trujillo et al. 2021 [53]

	MHC IIB exon 2<br>N=272<br>Environmental predictors	H_O = 0.00 - 0.86<br>AR = 1.0 – 6.5<br>T*D = -0.35 – 3.19*	Intensity: 40.7 – 35706 GE		increased risk of infection (P=0.03).<br>***Bd*** intensity was affected only by environm. variables: rainfall, temperature, latitude	Most measures of neutral and adaptive ***GD*** not correlated.<br>Adaptation to disease seems possible though selection on MHC genes.	
Atelopus varius & zeteki<br>Panama, TR<br>8 SS with declining or extinct pops, small N_E	Transcriptome sequencing , exome –capture assay to sequence coding regions<br>Decrease in genetic diversity<br>Pop. Structure K=8, no clear separation between *A. varius* and *zeteki* (but *zeteki* pops extinct)		Adult frogs<br>Survival after ***Bd*** infection	Fishers exact test	Immune related gene variants (outlier SNPs) associated with contemporary survivors after ***Bd*** caused declines (e.g. toll-like receptor genes)	The study uncovered candidate genes related to immune system and skin integrity. Admixture in one pop point to potential of genetic rescue	Byrne et al. 2021 [69]
Six species<br>Brasil, TR<br>3 forest specialists<br>*Aplastodiscus leucopygius*<br>*Ischnocnema henselii*<br>*Boana semmilineata*<br>& 3 habitat generalists<br>*Dendrosophus minutus*<br>*Boana polytaenia*<br>*Dendrosophus branneri*<br>6 areas, 2SS / species	MHC IIB Exon2 genotyping;<br>Genomic data: SNPs (ddRAD seq.)	N_A, H_E , H_O, π<br>Indiv: heterozygosity<br>Lower MHC H_E in fragmented forest compared to continuous forest; MHC diversity was inversely related to ddRAD diversity<br><br>NO DATA FOR SINGLE SPECIES	Adult frogs<br>***Bd*** infection	GLMs,<br>T-Test,<br>Chi^2-Test	Reduced pop level MHC diversity (H_E , H_O, but not π) correlated with increased ***Bd*** infection; no correlation with neutral genetic diversity (ddRAD); MHC IIB heterozygote indiv. tended to be less infected than homozygotes (n.s.)	***Bd*** prevalence highest in fragmented forest and forest specialist; Habitat fragmen-tation increases ***Bd*** infection susceptibility mediated through erosion of immunogenetic diversity	Belasen et al. 2022 [34]
Eleutherodactylus coqui<br>Puerto Rico, TR<br>1 SS,<br>N_c = 141;<br>Bottlenecked pop.<br>Probably in decline	Genomic data: SNPs (GBS)<br>n = 77<br>Anti-***Bd*** bacterium<br>Microbiome	F_{IS}: 0.04-0.72<br>HR: 0.57-4.38	Adults frogs<br>***Bd*** infection	Structural equation models	Frogs with low HR were more likely to be infected; no correlation between microbiome diversity and HR<br>Abundance of anti-***Bd*** bacterium higher in infected frogs	Offsetting effect: frogs with higher HR were less likely to be infected; but frogs with higher HR and infected frogs showed higher bacterium abundance; study highlights importance to study multiple interactions	Torres-Sanchez & Longo 2022 [66]

Abbreviations: **SS** = sample site, **TE** = Temperate region, **ST**= Subtropical region, **TR** = Tropical region, **n** = no. of individual investigated, **Pop** = population, **Tad** = tadpoles, **K** = no. of genetic cluster according to Bayesian assignment analysis (pop. Structure); **N** = pop size; $\mathbf{N_C}$= census pop size, $\mathbf{N_E}$ = effective pop size, ***Bd*** = Chytrid fungus (***B****atrachochytrium* ***d****endrobatidis*); **pw** = pairwise

Genetic diversity (***GD***) estimates: **P** = % polymorphic loci, **Na/l =** no. of alleles /locus, $\mathbf{H_E}$ = expected heterozygosity, $\mathbf{H_O}$ = observed heterozygosity, **H** = individual heterozygosity, $\mathbf{F_{IS}}$ **=** inbreeding coefficient; mean $\mathbf{d^2}$ = squared distance (in repeat units) between the two alleles within a locus, averaged over all loci analysed in an individual (Coulson et al. 1998); $\mathbf{H_{EST}}$ and $\mathbf{d^2_{EST}}$ estimated from parental genotypes, $\mathbf{r_{xy}}$, a pairwise relatedness estimator which measures the relatedness of the parents used in the common garden experiment (Queller & Goodnight 1989), **AR** = allelic richness, **HS** = gene diversity (microsats), **Npa** = no. of private alleles, $\mathbf{F_{ST}}$ = fixation index (population structure), **MLH** = indiv. multilocus heterozygosity, **IR** = ind. internal relatedness, $\mathbf{N_A}$ = allelic diversity (MHC alleles), **GSI** = Gini-Simpson Index (RAPD), **Nh** = Number of haplotypes, **h** = haplotype diversity, **π** = nucleotide diversity, **NE** = Effective number of alleles, **T*D*** = Tajima's D, **HR** = Heterozygous/homozygous ratio, **HFC** = heterozygosity – fitness correlation, **GFC** = Genetic variability fitness correlations
